# Self-reported and measured weights and heights among adults in Seattle and King County

**DOI:** 10.1186/s40608-016-0088-2

**Published:** 2016-02-18

**Authors:** Wesley Tang, Anju Aggarwal, Anne Vernez Moudon, Adam Drewnowski

**Affiliations:** Center for Public Health Nutrition, University of Washington, Box 353410, Seattle, WA 98195 USA; Urban Form Lab, University of Washington, 1107 NE 45th St, Seattle, WA 98105 USA

**Keywords:** Obesity, Anthropometry, Body mass index, Weight, Height

## Abstract

**Background:**

Self-reported weights and heights can be subject to gender, socio-economic, and other biases. On the other hand, obtaining measured anthropometric data can pose a significant respondent burden.

**Methods:**

Seattle Obesity Study II (SOS II) participants (*n* = 419) provided self-reported height, weight, and demographic data through an interviewer-assisted behavior survey. Participants were then weighed and measured by trained staff. The entire process was repeated 12 months later. At the follow up visit, participants were also asked to recall their weight from 12 months ago. The concordance between measured and self-reported data was assessed using Bland-Altman plots.

**Results:**

Some weight underreporting by obese individuals was observed. Gender or socio-economic status (SES) did not affect self-reports. Bland-Altman plots provided 95 % limits of agreement of −3.13 to 5.83 for weight (kg), and 1.21 to 2.52 for BMI (kg/m^2^). The concordance between measured and self-reported BMI categories was excellent (Kappa = 0.82 for men, and 0.86 for women). At the follow up visit, participants estimated their weight 12 months ago more accurately than their current weight.

**Conclusions:**

Self-reported heights and weights were highly correlated with objective measures at two points in time. No gender or SES biases were observed. Minor, yet statistically significant under-reporting (<1.5 kg) was observed for obese participants. Caution should be used when using self-reported data in obese populations.

**Electronic supplementary material:**

The online version of this article (doi:10.1186/s40608-016-0088-2) contains supplementary material, which is available to authorized users.

## Background

Adult obesity in the United States is a public health problem [1], with more than one-third of the adult population classified as obese [2]. Given multiple links to hypertension, cardiovascular disease [3], and cancer [4], body weight surveillance at the population-level is a matter of public health concern.

Objective anthropometric measures of height and weight, obtained using trained staff and standardized equipment, are the most commonly gathered metrics [5]. While the method of choice in clinical research, objective measures are less practical in state- or county-wide population-based surveys. Geographic distances between researchers and study participants, and the cost and time needed to gather relevant data have proven to be formidable barriers. Whereas the National Health and Nutrition Examination Survey (NHANES) collects measured heights and weights in the course of mobile clinic visits [6], the federal Behavioral Risk Factors Surveillance System (BRFSS) relies on heights and weights collected through telephone self-report [7].

The accuracy of self-reported heights and weights has been the focus of multiple prior studies [8–11]. Social desirability may be one reason why women tend to underreport their weight [12], more so than men [10]. Both men and women tend to over-report their height [12, 13]. Socio-economic status (SES) may be another source of reporting bias. Some studies have observed an effect of SES in self-reports [13–15]. For example, individuals in low SES underreported their weight and BMI to a greater extent than those with high SES [16, 17], whereas one study did not find an association between SES and reporting bias [18].

To the author’s knowledge, no study has been repeated on the same subjects at multiple time points. The present study is unique in that this study compared measured and self-reported heights and weights in a representative and geographically distributed longitudinal cohort of adults, separated by 12 months. At the follow up visit, study participants were asked to report their weight from 12 months ago as well as their current weight. Analyses explored the concordance between reported and observed measures and any potential bias due to gender, SES, or weight status. The goal was to determine whether self-reported data could provide a reasonably accurate estimate of population body weight at a much lower burden to respondents.

## Methods

### The Seattle obesity study II (SOS II) - sampling and recruitment

The Seattle Obesity Study II (SOS II) was a population-based prospective cohort study of adult residents of King County, WA, conducted in 2011–2013. An address-based sampling scheme stratified by property values was used to obtain a broad distribution of respondents by geographic location, race/ethnicity, and socioeconomic status (SES). A total of 17,500 addresses matched to telephone numbers were sent to the vendor, Battelle Memorial Institute. Pre-notification postcards mailed by the vendor identified the research as being conducted by the University of Washington. A week after receiving pre-notification postcards, the vendor called the phone numbers associated with sampled households. Three attempts were made to reach each potential respondent in the sampling frame. Upon contact with the household, addresses and telephone numbers were confirmed with respondents, along with eligibility criteria that respondents were 18–55 years, were English speakers, and had no mobility problems. A standard recruitment script was read, and a verbal consent was given over the phone. Interested and eligible household information was then passed to University of Washington SOS II staff.

During the following weeks, SOS II staff called identified households, reconfirmed interest and eligibility, and proceeded to schedule in-person interviews. Telephone calls by SOS II staff were placed throughout the day, with up to fifteen calls made per household.

### SOS II—data collection

Interviews were conducted at the date, time, and place of a participant’s choice. Participants completed a computer-assisted health and behavior survey in the course of a face to face interview. During the interview, participants were asked to self-report their socio-economic status and educational attainment levels using categorical options. For example, cut offs for income categories were chosen at < $50,000, $50,000–< $100,000, and ≥ $100,000 based on prior work with the county’s demographics.

Additionally, participants reported their heights and weights knowing that they would be weighed and measured following the completion of the survey, as specified in the previously signed consent form. Participants were not given documentation of the anthropometric measurements taken at baseline. BMI categorizations were calculated from self-reported height and weight values following established standard classification, < 18.5 Underweight, 18.5 to 24.9 Normal, 25.0 to 29.9 Overweight, >30.0 Obese.

### SOS II follow Up

The survey and anthropometric measurements were repeated at 12 months from baseline with reminder calls given at 3, 6, and 9 month intervals. Participants who completed the first meeting, did not become pregnant over the course of the last 12 months, and who still lived in King County, WA, were invited for a 2nd meeting. Subjects were also asked to recall their weight from 12 months ago. The study protocol was approved by the institutional review board (IRB) at the University of Washington. Informed consent was obtained from each participant during recruitment at both baseline and at 12 mo. follow up.

### Measurements

Measured weights were taken with a LifeSource Precision Scale Model UC-321 and measured heights were taken with a Charder HM200P Portstad portable stadiometer by a trained interviewer. Weights were recorded in pounds and height recorded in inches, to be later converted to metric units.

### Statistical analysis

Using measured and self-reported weight (kg), along with measured and self-reported height (m), measured and self-reported body mass index (BMI) was calculated (kg/m^2^). Participant demographics were summarized with descriptive statistics. Differences between measured and self-reported covariates were compared using paired t-tests. Differences between measured weight and recalled weight were also compared using paired t-tests. Positive differences represented an underreporting of a value whereas negative differences represented over-reporting of a value. Linear regression was also conducted to assess trends within a SES category. Cohen’s kappa coefficient was used to measure the concordance between measured and self-reported BMI categories. This assessment was done both for overall and by gender. Bland-Altman analyses were conducted to visually examine the degree of agreement between measured and self-reported anthropometric measurements. For these analyses, a discrepancy between the difference in measured minus self-reported is plotted against the mean of the two measured values. The limits of agreement were calculated as the mean difference between the two measurements ±1.96 times the standard deviation. All analyses were conducted using Stata 13.1 (College Station, TX), *p* < 0.05 was considered significant.

## Results

The distribution of SOS II participants is summarized in Table 1. Out of 419 participants, 135 were men and 293 were women. Most participants were <49 years old (60.1 %), white (84.4 %), and college graduates (63.8 %). Because only 3 individuals in the study sample were classified as ‘underweight’ using measured BMI at baseline, the categories of ‘underweight’ and ‘normal’ BMI have been combined. The combination of categories did not change the present results.

**Table 1:** Seattle Obesity Study II sample distributions

	n (%)
Overall	419
Age, years	
21-49	253 (60.4)
≥ 50	166 (39.6)
Gender	
Women	286 (68.3)
Men	133 (31.7)
Race/Ethnicity	
White	353 (84.5)
Non-White	65 (15.5)
Highest Education	
≤ Some college	150 (35.8)
College graduates	269 (64.2)
Annual Household Income	
< $50,000	116 (27.7)
$50,000–< $100,000	154 (36.8)
≥ $100,000	149 (35.6)
BMI	
Underweight or Normal	160 (38.2)
Overweight	117 (27.9)
Obese	142 (33.9)
Overall Measured Values	
Weight (kg)	81.17 (21.54)
Height (m)	1.69 (0.09)
BMI (kg/m^2^)	28.25 (6.85)
Overall Self-Reported Values	
Weight (kg)	79.82 (21.42)
Height (m)	1.70 (0.10)
BMI (kg/m^2^)	27.60 (6.59)

Table 2 shows mean measured and self-reported body weight at baseline. There was minor but systematic underreporting of body weight that was observed across all levels of age, gender, race/ethnicity, education, income, and BMI status. On the average, self-reported weights were lower by 1.35 kg (95 % CI 1.13,1.56) than were measured weights. For example, women underreported their weight by 1.49 kg (95 % CI 1.24,1.74), whereas men underreported their weight by 1.03 kg (95 % CI 0.63,1.48). These differences between men and women were not statistically significant from one another (*p* = 0.067).

**Table 2:** Comparisons between measured and self-reported weight at baseline

	Baseline measured weight (kg)	Baseline reported weight (kg)				
	Mean (SD)	Mean (SD)	Difference^a^	95 % CI	*P*-Value^b^	P for trend^c^
Overall	81.17 (24.54)	79.82 (21.42)	1.35	(1.13,1.56)	<0.001	
Age						
21–49	80.41(21.62)	79.04(21.42)	1.37	(1.09,1.64)	<0.001	
≥ 50	82.32(21.45)	81.01(21.45)	1.31	(0.96,1.67)	<0.001	0.821
Gender						
Men	90.67(21.32)	89.64(21.75)	1.03	(0.61,1.46)	<0.001	
Women	76.74(20.21)	75.25(19.70)	1.49	(1.24,1.74)	<0.001	0.067
Race/Ethnicity						
White	81.54(21.74)	80.22(21.74)	1.33	(1.10,1.56)	<0.001	
Non-white	79.11(20.46)	77.66(19.86)	1.45	(0.80,2.09)	<0.001	0.722
Highest education						
≤ Some college	86.35(24.70)	84.62(24.60)	1.73	(1.24,2.22)	<0.001	
College graduates	78.27(19.01)	77.15(18.96)	1.13	(0.93,1.33)	<0.001	**0.024**
Annual household income						
< $50,000	86.87(24.53)	85.05(24.57)	1.82	(1.24,2.41)	<0.001	
$50,000–< $100,000	81.70(20.46)	80.51(20.38)	1.19	(0.86,1.52)	<0.001	
≥ $100,000	76.18(18.97)	75.04(18.79)	1.13	(0.91,1.36)	<0.001	**0.031**
BMI						
Underweight or normal	63.36(8.51)	62.51(8.57)	0.86	(0.64,1.07)	<0.001	
Overweight	80.21(10.82)	78.74(10.89)	1.47	(1.11,1.84)	<0.001	
Obese	102.01(20.00)	100.22(20.50)	1.79	(1.28,2.30)	<0.001	**0.001**

^a^Difference = Difference between measured and self-reported weight

^b^ *P*-value = from paired *t*-test of mean difference (measured-self reported)

^c^ *P*-value = from linear regression comparing mean difference across a category, *p*-values <0.05 are in bold

Greater underreporting at baseline was associated with lower education and incomes. College-educated participants underreported by 1.13 kg, whereas those lacking college education underreported by 1.73 kg (*p* = 0.024). Obese participants underreported weight by 1.79 kg, as compared to only 0.86 kg for normal weight (*p* = 0.001).

Table 3 shows self-reported and measured body weight data obtained during the 12 mo. follow up. Again, there was a minor but systematic underreporting of body weight, calculated at 0.97 kg (95 % CI 0.74, 1.21). At follow up, there were no effects of either education or income on differences in reporting. However, obese participants underreported weight by 1.47 kg, as compared to only 0.48 kg for normal weight (*p* = 0.001).

**Table 3:** Comparisons between measured and self-reported weight at 12 months follow up

	12-month follow up measured weight (kg)	12-month follow up reported weight (kg)				
	Mean (SD)	Mean (SD)	Difference^a^	95 % CI	*P*-value^b^	P for trend^c^
Overall	80.61(21.56)	79.64(21.40)	0.97	(0.74,1.21)	<0.001	
Age						
21–49	80.06(21.45)	79.25(21.42)	0.81	(0.48,1.15)	<0.001	
≥ 50	81.45(21.76)	80.23(21.43)	1.22	(0.91,1.53)	<0.001	0.077
Gender						
Men	90.37 (21.19)	89.52 (21.43)	0.85	(0.37,1.33)	<0.001	
Women	76.07 (20.22)	75.04 (19.81)	1.03	(0.77,1.30)	<0.001	0.507
Race/Ethnicity						
White	81.03 (21.66)	80.00 (21.62)	1.04	(0.81,1.26)	<0.001	
Non-white	78.33 (21.00)	77.68 (20.22)	0.64	(−0.29,1.57)	0.172	0.411
Highest education						
≤ Some college	85.43 (24.41)	84.31 (24.41)	1.12	(0.64,1.61)	<0.001	
College graduates	77.92 (19.32)	77.03 (19.08)	0.89	(0.64,1.14)	<0.001	0.410
Annual household income						
< $50,000	86.01 (23.99)	84.94 (23.85)	1.08	(0.50,1.65)	<0.001	
$50,000–< $100,000	80.94 (21.00)	79.86 (20.87)	1.08	(0.72,1.44)	<0.001	
≥ $100,000	76.07 (19.12)	75.28 (18.99)	0.79	(0.46,1.11)	<0.001	0.355
BMI						
Underweight or normal	63.49 (8.61)	63.01 (8.49)	0.48	(0.20,0.76)	<0.001	
Overweight	80.20 (10.00)	79.05 (10.25)	1.15	(0.68,1.62)	<0.001	
Obese	103.93 (20.08)	102.46 (20.58)	1.47	(0.96,1.97)	<0.001	**0.001**

^a^Difference = Difference between measured and self-reported weight

^b^ *P*-value = from paired *t*-test of mean difference (measured-self reported)

^c^ *P*-value = from linear regression comparing mean difference across a category, *p*-values <0.05 are in bold

Table 4 shows measured weight at baseline and subject recall of baseline weight 12 months later. The mean underestimate (measured weight—recalled weight) was 0.90 kg (95 % CI 0.51, 1.30). There was no bias in recall by age, gender, race/ethnicity, education, income, or obesity status.

**Table 4:** Comparisons between measured weight at baseline and participant recall of baseline weight at 12 months follow up

	Baseline measured weight (kg)	Recalled reported weight (kg)				
	Mean (SD)	Mean (SD)	Difference^a^	95 % CI	*P*-value^b^	P for trend^c^
Overall	81.17 (21.54)	80.26 (21.84)	0.90	(0.51,1.30)	<0.001	
Age						
21–49	80.41 (21.62)	79.46 (21.62)	0.95	(0.46,1.43)	<0.001	
≥ 50	82.32 (21.45)	81.48 (22.20)	0.84	(0.16,1.52)	0.016	0.804
Gender						
Men	90.67 (21.32)	90.04 (22.11)	0.64	(−0.37,1.64)	0.212	
Women	76.74 (20.21)	75.72 (20.20)	1.03	(0.68,1.38)	<0.001	0.470
Race/Ethnicity						
White	81.54 (21.74)	80.64 (21.12)	0.91	(0.49,1.33)	<0.001	
Non-white	79.11 (20.46)	78.22 (20.33)	0.89	(−0.27,2.04)	0.130	0.976
Highest education						
≤ Some college	86.35 (24.70)	85.36 (24.49)	0.99	(0.25,1.74)	0.010	
College graduates	78.27 (19.01)	77.42 (19.70)	0.86	(0.39,1.32)	<0.001	0.761
Annual household income						
< $50,000	86.87 (24.53)	85.93 (24.20)	0.94	(0.06,1.83)	0.038	
$50,000–< $100,000	81.70 (20.46)	80.91 (21.53)	0.78	(0.02,1.54)	0.043	
≥ $100,000	76.18 (18.97)	75.18 (19.03)	1.00	(0.58,1.42)	<0.001	0.876
BMI						
Underweight or normal	63.36 (8.51)	62.56 (8.61)	0.80	(0.36,1.24)	<0.001	
Overweight	80.21 (10.82)	79.21 (12.49)	1.00	(0.08,1.93)	0.034	
Obese	102.01 (20.00)	101.07 (20.25)	0.94	(0.18,1.69)	0.015	0.745

^a^Difference = Difference between measured weight at baseline and recalled weight

^b^ *P*-value = from paired *t*-test of mean difference (measured-recalled)

^c^ *P*-value = from linear regression comparing mean difference across a category

### Bland-Altman plots of weight and BMI

**Fig. 1:**
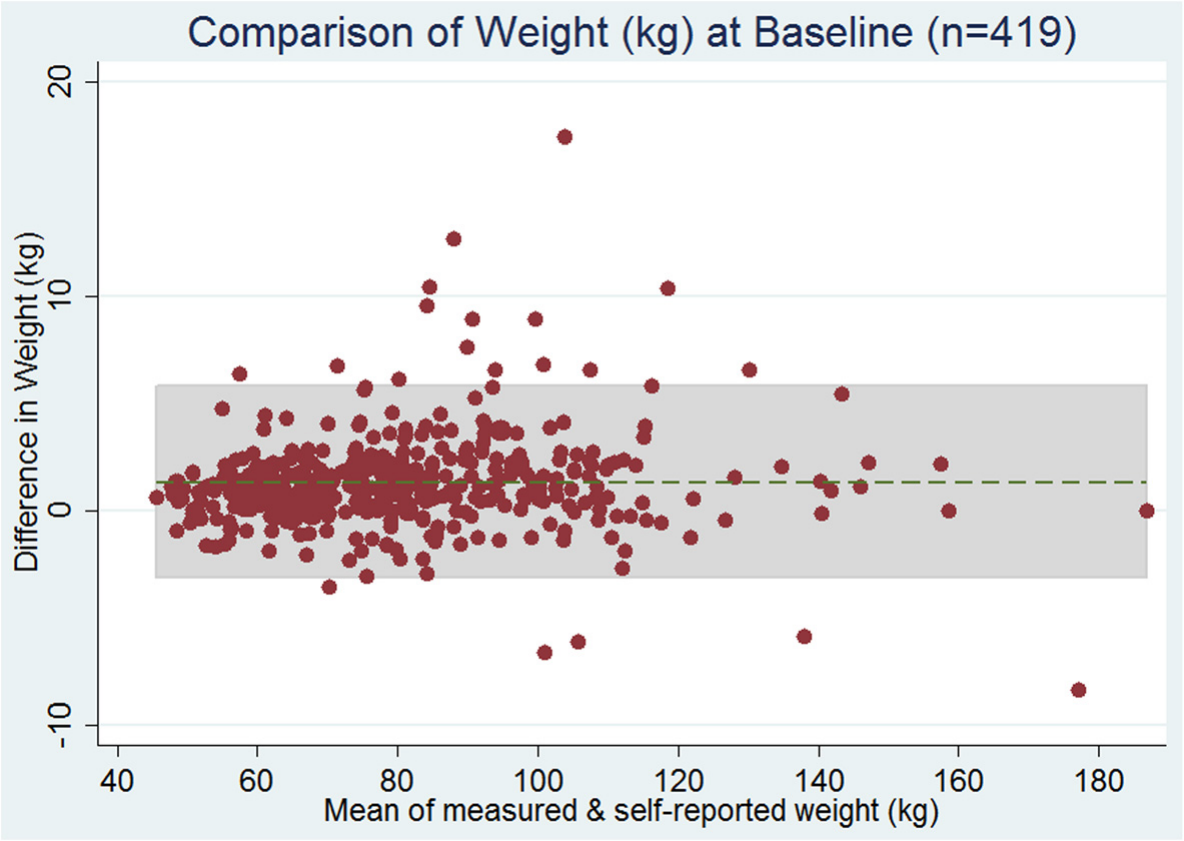
Overall Bland-Altman plots of the difference in weight vs. mean of measured and self-reported weight (kg) at baseline

Overall, high agreement was observed between measured weight (kg) and self-reported weight (kg), at baseline (Fig. 1). The limits of agreement in the differences of weight ranged from −3.13 to 5.83.

**Fig. 2:**
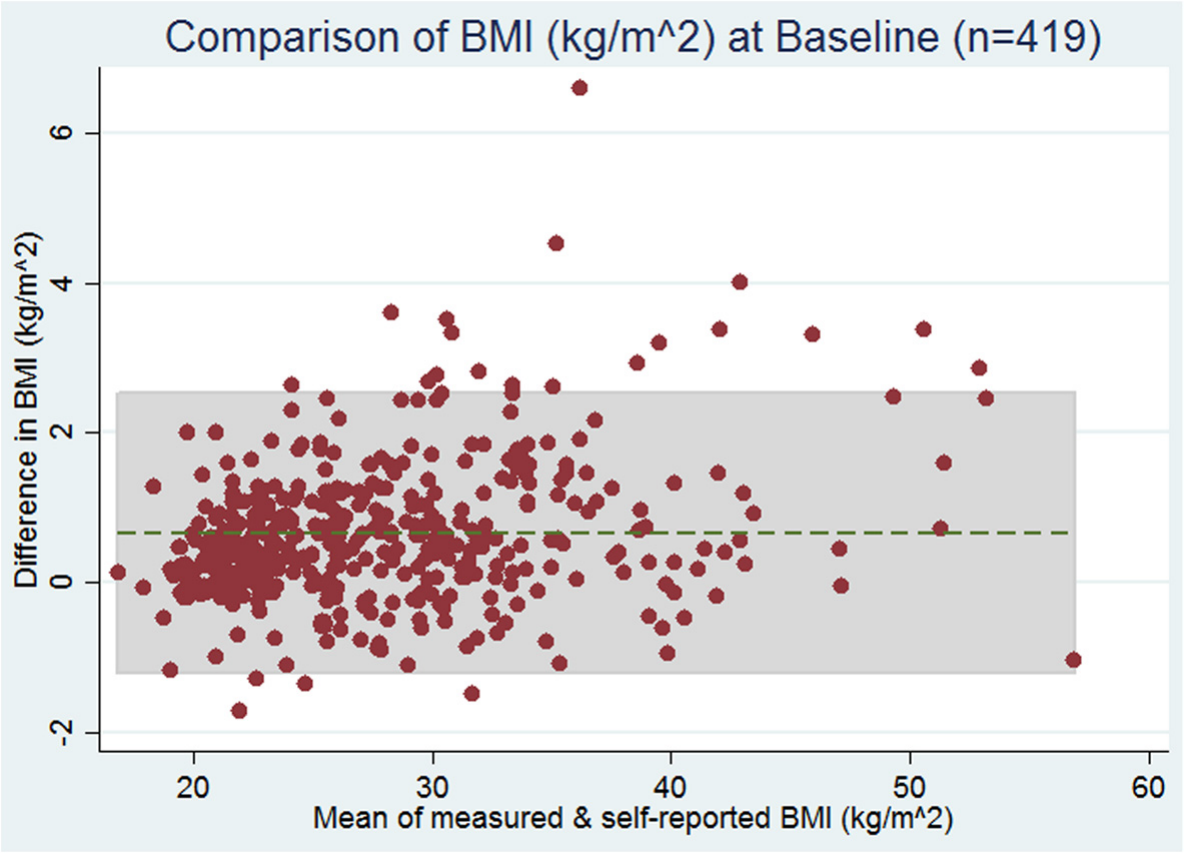
Overall Bland-Altman plots of the difference in BMI vs. mean of measured and self-reported BMI (kg/m^2^) at baseline

Like weight, high agreement was observed between measured BMI (kg/m^2^) and self-reported BMI, at baseline (Fig. 2). The limits of agreement in the differences of BMI ranged from −1.21 to 2.52.

### Concordance of BMI categories

The degree of agreement across measured and self-reported BMI categories at baseline is presented in Table 5. The overall agreement assessed using kappa statistics, was 90.2 % (κ = 0.85, *p* < 0.0001). High values of agreement were also observed by gender (κ = 0.82 for males, *p* < 0.0001, κ = 0.86 for females, *p* < 0.0001). These kappa coefficient values indicate excellent agreement between measured and self-reported BMI categories.

**Table 5:** Concordance of baseline BMI, measured vs. self-report

	Self-reported BMI category					
	Underweight/normal weight	Overweight	Obese	Agreement	Kappa	*p*-value
	n (%)	n (%)	n (%)			
Measured BMI category						
Overall						
Underweight/normal weight	158 (98.8)	2 (1.3)	0 (0)			
Overweight	17 (14.5)	99 (84.6)	1 (0.9)	90.2 %	0.85	**<0.0001**
Obese	0 (0)	21 (14.8)	121 (85.2)			
Gender						
Male						
Underweight/normal weight	44 (97.8)	1 (2.2)	0 (0)			
Overweight	7 (15.6)	38 (84.4)	0 (0)	88.0 %	0.82	**<0.0001**
Obese	0 (0)	8 (18.6)	32 (81.4)			
Female						
Underweight/normal weight	114 (99.1)	1 (0.9)	0 (0)			
Overweight	10 (13.9)	61 (84.7)	1 (1.4)	91.3 %	0.87	**<0.0001**
Obese	0 (0)	13 (13.1)	86 (86.9)			

*p*-values <0.05 are in bold

Similar analyses were conducted for calculated BMI values at 12 months follow up (data not shown). The overall agreement was 91.4 % (κ = 0.87, *p* < 0.0001). High values of agreement were also observed by gender (κ = 0.83 for males, *p* < 0.0001, κ = 0.88 for females, *p* < 0.0001). These kappa coefficient values indicate excellent agreement between measured and self-reported BMI categories.

### Additional comparisons

Additional file 1: Table S1 shows analyses of measured and self-reported height at baseline. Overall, subjects over-reported their height by 0.48 cm (*p* < 0.0001). Men over-reported their height by 1.13 cm (*p* < 0.0001). Women over-reported their height by 0.14 cm (NS; *p* = 0.06).

Additional file 2: Table S2 shows analyses of measured and self-reported height at 12mo. follow up. Overall, subjects over-reported their height by 0.35 cm (*p* < 0.0001). Men over-reported their height by 0.80 cm (*p* < 0.0001). Women over-reported their height by 0.14 cm (NS; *p* = 0.11).

Additional file 3: Table S3 shows a comparison of measured and self-reported BMI at baseline. Obese participants underreported BMI by 1.01 units; overweight participants underreported by 0.6 units, and normal weight underreported by 0.36 units (*p* < 0.001). A similar trend was observed at the 12 months follow up (*p* < 0.001) (Data not shown).

Additional file 4: Figure S1 shows a Bland-Altman plot comparing measured and self-reported heights at baseline. Overall, high agreement was observed. The limits of agreement in the differences of height ranged from −3.90 to 2.94.

## Discussion

Overall measured anthropometric data was closely tracked by self-reported anthropometric data. While differences in weight were statistically significant, differences were not observed to be higher than 1.8 kg. For height, differences were also statistically significant, with subjects over-reporting heights by 0.48 cm. Overall measures via kappa coefficients showed excellent agreement, and Bland-Altman plots produced narrow limits of agreement. These observations held true for the same sample at baseline and at 12 months follow up.

As in previous studies, this sample of adult residents of King County, WA showed that participants are underreporting their weight. The overall mean discrepancy of 1.35 kg is comparable to other studies’ bias [11] and also when stratified by gender [19]. Underreporting is especially pronounced in individuals of higher BMI categories. For example, the difference between the measures for weight in obese individuals is 0.93 kg higher than for underweight/normal individuals. This may be observed because obese individuals may feel a social pressure to report lower weights as compared to individuals who are underweight/normal weight status. This may also be observed because individuals who are obese may have their weight fluctuate more than individuals of other categories, leading to higher differences between measured and self-reported weight.

There does not appear to be a large, or consistent effect of socio-economic status on weight estimation. While college graduates were observed to have a difference between measured & self-reported weight 0.60 kg lower than ≤ some college individuals, this relationship was not statistically significant at 12 months follow up. Similarly, this trend exists at baseline for income but not at 12 months follow up. There were no differences by socio-economic status on weight estimation by age, gender, or race/ethnicity at baseline or at 12 months follow up. When comparing the present study to previous studies that did observe a reporting bias by SES, two possibilities emerge. The first is that bias in self-reporting anthropometry by SES may truly be different depending on differing populations. Another possibility for not observing an effect by SES may be because of the characteristics of King County, WA which has a higher than national average annual household income and educational attainment.

Oddly, subjects more closely recalled their weight from 12 months in the past than when they were originally asked to self-report their weight. This provides evidence that not only can respondents accurately self-report their present weight, they can also accurately self-report their weight from the past. As for why the difference in recall to measured is smaller than self-reported to measured, this cannot be easily explained. One possible explanation is that subjects became more aware of their weight in the period from baseline to 12 month follow up.

This study had several limitations. First, participants were informed ahead of time that research staff would be measuring anthropometric data at the end of a study session. This likely encourages participants to report more accurate values than had they not been informed. Second, there will be variability to the difference in a person’s measured and self-reported weight & BMI depending on the time of day and foods and beverages consumed. Whereas a person’s self-reported weight may not change throughout a day, a person’s measured weight will. Third, the sampled population is over-representative of white, college-educated, women, from the Seattle/King County, WA, USA area. These demographics are not reflective of the entire US population and the results may not be generalizable to a different population.

## Conclusions

Consistent with previous studies, the present study provides evidence that self-reported height and weight may be used as a proxy for measured height and weight. Although biased and underreporting by 1.35 kg, individuals in different socio-economic groupings did not appear to consistently estimate their weight differently from one another. Likewise, subjects were able to accurately recall their weight from 12 months prior with differences measured at only 0.90 kg. As self-reporting can cost a fraction of the amount necessary compared to measurements, self-reporting should be considered a potential option in assessing anthropometry. Caution should be taken in applying these methods to overweight and obese populations.

## Additional files

Additional file 1: Table S1. Comparisons between measured and self-reported height at baseline. (DOCX 16.5 kb) Additional file 2: Table S2. Comparisons between measured and self-reported height at 12mo. follow up. (DOCX 16.5 kb) Additional file 3: Table S3. Comparisons between measured and self-reported BMI at baseline. (DOCX 16.4 kb) Additional file 4: Figure S1. Comparison of Height (cm) at Baseline (n=419). (TIF 2.49 mb)
