# Supplementary material for: Self-reported and measured weights and heights among adults in Seattle and King County
Source: BMC Obes. 2016 Feb 18;3:11. doi: 10.1186/s40608-016-0088-2 (PMC4757992; doi:10.1186/s40608-016-0088-2)
Supplement: Additional file 1: Table S1. — Comparisons between measured and self-reported height at baseline. (DOCX 16.5 kb) [file 40608_2016_88_MOESM1_ESM.docx]

Table S1: Comparisons between measured and self-reported height at baseline

|  |  | **Baseline Measured Height (cm)** | **Baseline Reported Height (cm)** |  |  |  |  |
| --- | --- | --- | --- | --- | --- | --- | --- |
|  |  | **Mean (SD)** | **Mean (SD)** | **Difference^a^** | **95% CI** | **P-Value^b^** | **P for trend^c^** |
| **Overall** | | 169.28 (9.23) | 169.76 (9.66) | -0.48 | (-0.65,-0.311) | <0.0001 |  |
| **Age** | |  |  |  |  |  |  |
|  | 21-49 | 169.76 (9.12) | 170.28 (9.48) | -0.52 | (-0.74,-0.30) | <0.0001 |  |
|  | ≥50 | 168.54 (9.37) | 168.95 (9.91) | -0.41 | (-0.67,-0.15) | 0.0022 | 0.508 |
| **Gender** | |  |  |  |  |  |  |
|  | Men | 178.67 (6.68) | 165.08 (6.81) | -1.13 | (1.91,-1.46) | <0.0001 |  |
|  | Women | 164.91 (6.62) | 179.80 (6.84) | -0.18 | (-0.36,0.01) | 0.0615 | <0.001 |
| **Race/Ethnicity** | |  |  |  |  |  |  |
|  | White | 170.00 (9.26) | 170.40 (9.68) | -0.41 | (-0.58,-0.23) | <0.0001 |  |
|  | Non-White | 165.35 (8.00) | 166.23 (8.81) | -0.87 | (-1.40,-0.35) | 0.0015 | 0.091 |
| **Highest Education** | |  |  |  |  |  |  |
|  | ≤ Some college | 167.60 (9.01) | 168.37 (9.57) | -0.77 | (-1.08,-0.46) | <0.0001 |  |
|  | College graduates | 170.21 (9.23) | 170.53 (9.65) | -0.32 | (-0.51,-0.12) | 0.0016 | 0.015 |
| **Annual Household Income** | |  |  |  |  |  |  |
|  | <$50,000 | 170.40 (8.91) | 171.17 (9.51) | -0.77 | (-1.14,-0.40) | 0.0001 |  |
|  | $50,000-<$100,000 | 169.03 (9.35) | 169.48 (9.69) | -0.46 | (-0.73,-0.19) | 0.0010 |  |
|  | ≥$100,000 | 168.66 (9.32) | 168.93 (9.69) | -0.27 | (-0.52,-0.02) | 0.0329 | 0.026 |
| **BMI** | |  |  |  |  |  |  |
|  | Underweight or Normal | 168.74 (8.66) | 168.97 (9.16) | -0.24 | (-0.51,0.03) | 0.0804 |  |
|  | Overweight | 171.10 (9.85) | 171.47 (10.33) | -0.37 | (-0.68,-0.05) | 0.0227 |  |
|  | Obese | 168.38 (9.16) | 169.23 (9.53) | -0.84 | (-1.14,-0.55) | <0.0001 | 0.003 |
| ^a^Difference = Difference between measured and self-reported height | | | |  |  |  |  |
| ^b^P-value = p-value from paired t-test of mean difference (measured -self reported) | | | |  |  |  |  |
| ^c^P-value = from linear regression comparing mean difference across a category | | | |  |  |  |  |
