# Supplementary material for: Self-reported and measured weights and heights among adults in Seattle and King County
Source: BMC Obes. 2016 Feb 18;3:11. doi: 10.1186/s40608-016-0088-2 (PMC4757992; doi:10.1186/s40608-016-0088-2)
Supplement: Additional file 2: Table S2. — Comparisons between measured and self-reported height at 12mo. follow up. (DOCX 16.5 kb) [file 40608_2016_88_MOESM2_ESM.docx]

Table S2: Comparisons between measured and self-reported height at 12mo. follow up

|  |  | **12-month Follow up Measured Height (cm)** | **12-month Follow up Reported Height (cm)** |  |  |  |  |
| --- | --- | --- | --- | --- | --- | --- | --- |
|  |  | **Mean (SD)** | **Mean (SD)** | **Difference^a^** | **95% CI** | **P-Value^b^** | **P for trend^c^** |
| **Overall** | | 169.36 (9.30) | 169.70 (9.61) | -0.35 | (-0.49,-0.20) | <0.0001 |  |
| **Age** | |  |  |  |  |  |  |
|  | 21-49 | 169.87 (9.16) | 170.21 (9.44) | -0.34 | (-0.52,-0.16) | 0.0004 |  |
|  | ≥50 | 168.57 (9.48) | 168.93 (9.83) | -0.36 | (-0.59,-0.13) | 0.0025 | 0.885 |
| **Gender** | |  |  |  |  |  |  |
|  | Men | 178.91 (6.77) | 179.71 (6.83) | -0.80 | (-1.07,-0.54) | <0.0001 |  |
|  | Women | 164.91 (6.57) | 165.05 (6.73) | -0.14 | (-0.30,0.03) | 0.1101 | <0.001 |
| **Race/Ethnicity** | |  |  |  |  |  |  |
|  | White | 170.03 (9.37) | 170.39 (9.63) | -0.36 | (-0.52,-0.21) | <0.0001 |  |
|  | Non-White | 165.69 (8.02) | 165.97 (8.61) | -0.28 | (-0.68,0.13) | 0.1742 | 0.705 |
| **Highest Education** | |  |  |  |  |  |  |
|  | ≤ Some college | 167.68 (8.99) | 168.26 (9.57) | -0.57 | (-0.84,-0.30) | <0.0001 |  |
|  | College graduates | 170.29 (9.36) | 170.51 (9.55) | -0.22 | (-0.39,-0.06) | 0.0084 | 0.030 |
| **Annual Household Income** | |  |  |  |  |  |  |
|  | <$50,000 | 170.51 (9.00) | 171.15 (9.49) | -0.64 | (-0.94,-0.34) | <0.0001 |  |
|  | $50,000-<$100,000 | 169.19 (9.31) | 169.39 (9.59) | 0.20 | (-0.42,0.03) | 0.0816 |  |
|  | ≥$100,000 | 168.63 (9.50) | 168.90 (9.66) | -0.28 | (-0.51,-0.04) | 0.0245 | 0.074 |
| **BMI** | |  |  |  |  |  |  |
|  | Underweight or Normal | 168.83 (8.71) | 168.99 (8.99) | -0.16 | (-0.40,0.08) | 0.1939 |  |
|  | Overweight | 171.09 (10.05) | 171.32 (10.28) | -0.23 | (-0.49,0.04) | 0.0936 |  |
|  | Obese | 168.52 (9.17) | 169.18 (9.61) | -0.66 | (-0.90,-0.42) | <0.0001 | <0.001 |
| ^a^Difference = Difference between measured and self-reported height | | | |  |  |  |  |
| ^b^P-value = p-value from paired t-test of mean difference (measured -self reported) | | | |  |  |  |  |
| ^c^P-value = from linear regression comparing mean difference across a category | | | |  |  |  |  |
