# Supplementary material for: Self-reported and measured weights and heights among adults in Seattle and King County
Source: BMC Obes. 2016 Feb 18;3:11. doi: 10.1186/s40608-016-0088-2 (PMC4757992; doi:10.1186/s40608-016-0088-2)
Supplement: Additional file 3: Table S3. — Comparisons between measured and self-reported BMI at baseline. (DOCX 16.4 kb) [file 40608_2016_88_MOESM3_ESM.docx]

Table S3: Comparisons between measured and self-reported BMI at baseline

|  |  | **Baseline Measured BMI (kg/m^2^)** | **Baseline Reported BMI (kg/m^2^)** |  |  |  |  |
| --- | --- | --- | --- | --- | --- | --- | --- |
|  |  | **Mean (SD)** | **Mean (SD)** | **Difference^a^** | **95% CI** | **P-Value^b^** | **P for trend^c^** |
| **Overall** | | 28.25 (6.85) | 27.60 (6.59) | 0.65 | (0.56,0.74) | <0.0001 |  |
| **Age** | |  |  |  |  |  |  |
|  | 21-49 | 27.82 (6.88) | 27.15 (6.60) | 0.67 | (0.55,0.78) | <0.0001 |  |
|  | ≥50 | 28.90 (6.76) | 28.28 (6.55) | 0.62 | (0.47,0.77) | <0.0001 | 0.631 |
| **Gender** | |  |  |  |  |  |  |
|  | Men | 28.37 (6.25) | 27.67 (6.13) | 0.70 | (0.53,0.87) | <0.0001 |  |
|  | Women | 28.19 (7.12) | 27.57 (6.81) | 0.62 | (0.51,0.73) | <0.0001 | 0.443 |
| **Race/Ethnicity** | |  |  |  |  |  |  |
|  | White | 28.14 (6.84) | 27.52 (6.59) | 0.62 | (0.52,0.71) | <0.0001 |  |
|  | Non-White | 28.84 (6.92) | 28.03 (6.67) | 0.81 | (0.51,1.10) | <0.0001 | 0.226 |
| **Highest Education** | |  |  |  |  |  |  |
|  | ≤ Some college | 30.55 (7.62) | 29.64 (7.30) | 0.92 | (0.72,1.11) | <0.0001 |  |
|  | College graduates | 26.96 (6.02) | 26.46 (5.88) | 0.50 | (0.41,0.59) | <0.0001 | <0.001 |
| **Annual Household Income** | |  |  |  |  |  |  |
|  | <$50,000 | 29.54 (7.62) | 28.82 (7.36) | 0.91 | (0.68,1.14) | <0.0001 |  |
|  | $50,000-<$100,000 | 28.55 (6.73) | 27.96 (6.46) | 0.60 | (0.46,0.74) | <0.0001 |  |
|  | ≥$100,000 | 26.69 (6.00) | 26.20 (5.83) | 0.49 | (0.38,0.61) | <0.0001 | 0.001 |
| **BMI** | |  |  |  |  |  |  |
|  | Underweight or Normal | 22.17 (1.63) | 21.81 (1.57) | 0.36 | (0.27,0.46) | <0.0001 |  |
|  | Overweight | 27.29 (1.46) | 26.68 (1.66) | 0.60 | (0.46,0.75) | <0.0001 |  |
|  | Obese | 35.88 (5.75) | 34.87 (5.65) | 1.01 | (0.80,1.21) | <0.0001 | <0.001 |
| ^a^Difference = Difference between measured and self-reported BMI | | | |  |  |  |  |
| ^b^P-value = p-value from paired t-test of mean difference (measured -self reported) | | | |  |  |  |  |
| ^c^P-value = from linear regression comparing mean difference across a category | | | |  |  |  |  |
