# Supplementary figures and images for: Self-reported and measured weights and heights among adults in Seattle and King County
Source: BMC Obes. 2016 Feb 18;3:11. doi: 10.1186/s40608-016-0088-2 (PMC4757992; doi:10.1186/s40608-016-0088-2)

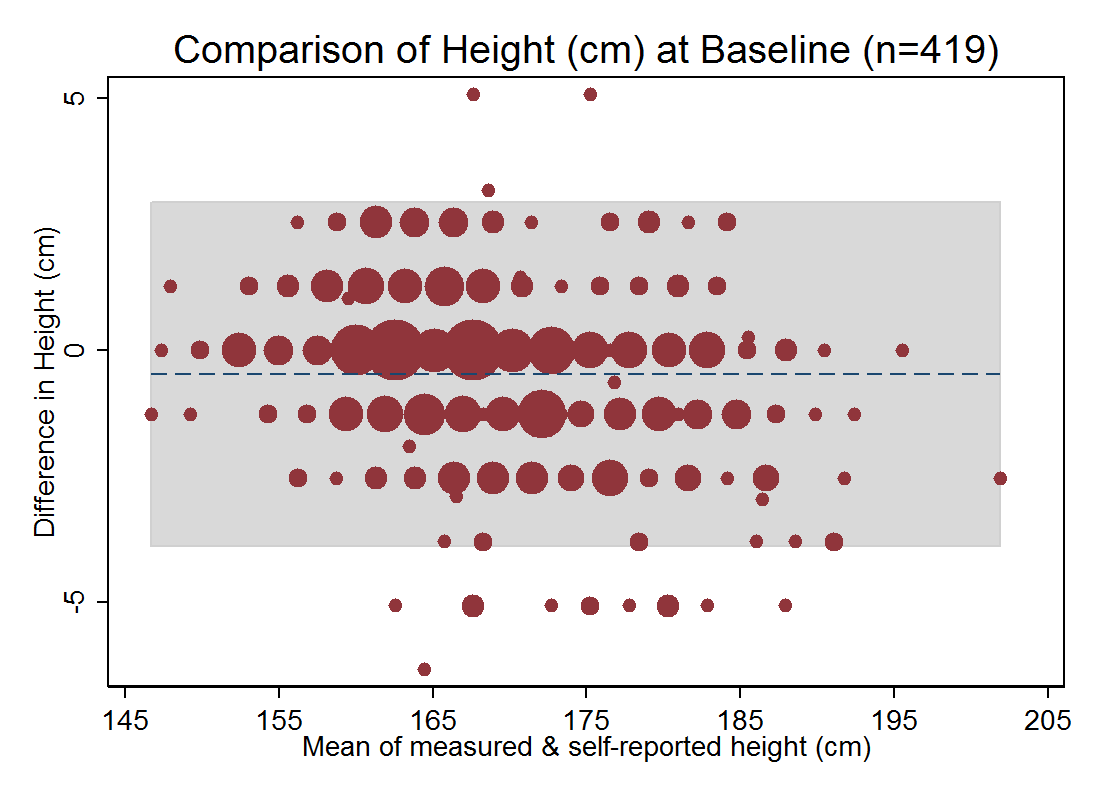

Supplement: Additional file 4: Figure S1. — Comparison of Height (cm) at Baseline (n=419). (TIF 2.49 mb) [file 40608_2016_88_MOESM4_ESM.tif]
